# Identification and Analysis of RNA Editing Sites in the Chloroplast Transcripts of *Aegilops tauschii* L.

**DOI:** 10.3390/genes8010013

**Published:** 2016-12-30

**Authors:** Mengxing Wang, Hui Liu, Lingqiao Ge, Guangwei Xing, Meng Wang, Song Weining, Xiaojun Nie

**Affiliations:** State Key Laboratory of Crop Stress Biology in Arid Areas, College of Agronomy, Northwest Agriculture and Forestry University, Yangling 712100, Xianyang, China; machine@nwsuaf.edu.cn (M.-X.W.); m15191417214@163.com (H.L.); lqiaoge@163.com (L.G.); fire_wind_withcity@163.com (G.X.); wm2008xs@163.com (M.W.)

**Keywords:** *Aegilops tauschii*, chloroplast, RNA editing, *Triticum*

## Abstract

RNA editing is an important way to convert cytidine (C) to uridine (U) at specific sites within RNA molecules at a post-transcriptional level in the chloroplasts of higher plants. Although it has been systematically studied in many plants, little is known about RNA editing in the wheat D genome donor *Aegilops tauschii* L. Here, we investigated the chloroplast RNA editing of *Ae. tauschii* and compared it with other wheat relatives to trace the evolution of wheat. Through bioinformatics prediction, a total of 34 C-to-U editing sites were identified, 17 of which were validated using RT-PCR product sequencing. Furthermore, 60 sites were found by the RNA-Seq read mapping approach, 24 of which agreed with the prediction and six were validated experimentally. The editing sites were biased toward tCn or nCa trinucleotides and 5′-pyrimidines, which were consistent with the flanking bases of editing sites of other seed plants. Furthermore, the editing events could result in the alteration of the secondary structures and topologies of the corresponding proteins, suggesting that RNA editing might impact the function of target genes. Finally, comparative analysis found some evolutionarily conserved editing sites in wheat and two species-specific sites were also obtained. This study is the first to report on RNA editing in *Aegilops tauschii* L, which not only sheds light on the evolution of wheat from the point of view of RNA editing, but also lays a foundation for further studies to identify the mechanisms of C-to-U alterations.

## 1. Introduction

RNA editing is a post-transcriptional process for converting cytidine (C) to uridine (U), or U to C, at specific sites within RNA molecules to alter the identity of nucleotides between RNA and genomic DNA, serving as a mechanism to correct missense mutations of genes at the RNA level, and also to enrich genetic information [1,2]. It can act upon transcripts in the cell nucleus, cytoplasm, mitochondria, and chloroplast (cp), which has been widely documented in various organisms, including eukaryotes, prokaryotes, archaebacteria, viruses, etc. [3,4,5]. Since the first editing event of the land plant plastome was found in the mRNA transcript of the *rpl2* gene in the maize chloroplast genome in 1991 [6], extensive RNA editing sites have been detected in a variety of higher plants, such as tobacco [7,8], *Arabidopsis* [9], pea [10], and tomato [11], and some of them have been validated by experiments [12]. Hornwort and fern exhibit hundreds of C-to-U as well as U-to-C editing sites [13,14]; however, in angiosperms, approximately 30 to 40 C-U editing sites are found in the chloroplasts [15]. In other land plant clades, editing frequencies vary widely, with no editing in marchantiid liverworts [16]. Although a few cases of editing within the noncoding regions or introns have been reported, most of the known editing events occur in exons, restoring corresponding amino acids to a more conserved direction [17,18]. Additionally, editing usually occurs in the second or first base of codons, resulting in the conversion of hydrophilic amino acid to hydrophobic, in most cases [19,20]. As an essential step of RNA maturation in plant organelles, the molecular mechanisms of RNA editing have been extensively studied [21]. It is widely accepted that PPR (pentatricopeptide repeat) proteins are the key factor to regulate RNA editing through binding to the target transcripts and then altering the RNA sequence turnover, processing, and translation [22,23,24].

*Aegilops tauschii* L., also known as Tausch’s goatgrass, is an annual grass belonging to the Triticeae tribe of the Poaceae family, which is found all over the world, including in China, Central and Western Asia, the Caucasus, Eastern Europe, and America. *Ae. tauschii* donated the D genome of hexaploid wheat (*Triticum aestivum* L.) by a spontaneous hybridization of the wild diploid grass *Ae. tauschii* with the tetraploid wheat *Triticum turgidum* about 8000–10,000 years ago in the Fertile Crescent [25,26], which holds promise for the underlying processes of wheat formation and evolution. Extensive studies have been conducted to reveal the roles of *Ae. tauschii* in the evolutionary origin of polyploid wheat from different viewpoints, such as complete genome sequencing [27], physical mapping [28], and plastome sequences [29], as well as population genetic analyses [30]. However, the RNA editing sites in *Ae. tauschii* have not been well understood up to now. To trace the evolutionary process of polyploid wheat from the perspective of chloroplast RNA editing, we investigated and compared the chloroplast RNA editing patterns of *Ae. tauschii* together with other wheat relative species in this study. Firstly, the RNA editing sites in 76 chloroplast transcripts of *Ae. tauschii* were identified using bioinformatics prediction, combined with the RNA-Seq read mapping approach. Then, these predicted editing sites were further validated through PCR and RT-PCR. Furthermore, the alterations of the secondary structures of these proteins were investigated to understand the potential biological functions of these editing events. Finally, the chloroplast RNA editing sites in *Ae. tauschii* were compared with those of common wheat, wheat A and B genome progenitors, as well as five other Poaceae species to provide information regarding the origin and evolution of chloroplast RNA editing in wheat and beyond.

## 2. Materials and Methods

### 2.1. Prediction of RNA Editing Sites

All of the 76 protein-coding genes in the plastome of *Ae. tauschii* (AL8/78) (GenBank Accession: KJ614412.1) were downloaded from the NCBI (National Center for Biotechnology Information) database (https://www.ncbi.nlm.nih.gov) according to their annotation information [29]. Potential RNA editing sites in these genes were predicted by the Predictive RNA Editor for Plants (PREP)-Cp web server (http://prep.unl.edu/cgi-bin/cp-input.pl) [31] with a cutoff value of 0.8. Bioedit [32] and Microsoft Excel tools were used to format the required input file.

### 2.2. Editing Site Detection by Read Mapping and SNP (Single Nucleotide Polymorphism) Calling

RNA-Seq data from eight tissues of *Ae. tauschii* (AL8/78) were downloaded from the Sequence Read Archive (SRA) database with the accession numbers of SRX209402~SRX209425 (Table S1) [27]. The RNA-Seq reads were mapped to the cp genome of *Ae. tauschii* (AL8/78) using the Tophat tool [33]. SNPs were called by SAMtools to detect potential changes of C to U in the sense-strand and G (guanine) to A (adenine) in the antisense-strand of the cp genome. The mapped reads were subsequently visualized in the Integrative Genomics Viewer [34]. Only the DNA:RNA mismatches that had at least 5% editing efficiency, and more than five individual read supports were kept. RNA editing efficiency was counted by edited reads divided by total mapped reads [2].

### 2.3. Protein Structure Analysis before and after Editing

CFSSP (Chou and Fasman Secondary Structure Prediction Server) (http://www.biogem.org/tool/chou-fasman/) [35,36] was used to analyze the protein secondary structures of amino acid sequences translated from edited and unedited RNA. Prediction of transmembrane segments before and after editing, was performed using the Phobius tool (http://phobius.sbc.su.se/) [37].

### 2.4. Comparative Analysis of RNA Editing Sites in Cp Protein-Coding Genes among Poaceae Species

Previous study has been conducted to reveal the origin and evolution of RNA editing in cereal mitochondria [38]. Similarly, we compared and analyzed RNA editing in chloroplasts of Poaceae species. Firstly, the RNA editing sites in cp protein-coding genes of *Ae. tauschii* were compared to those of *Hordeum vulgare* [39], *Lolium perenne* [40], *Oryza sativa* [41], *Saccharum officinarum* [42], and *Zea mays* [17] to identify the patterns of RNA editing in Poaceae plastomes. Furthermore, all of the chloroplast protein-coding genes of *Triticum urartu*, *Aegilops speltoides*, and *T. turgidum*, as well as *T. aestivum* (Table 1) [29], were downloaded and potential RNA editing sites in these genes were predicted using the methods described above, and then the differences among these species were investigated.

### 2.5. Validation of Partial RNA Editing Sites

Seeds of *Ae. tauschii* (AL8/78) were put on moistened filter paper in a dish and then cultured in a growth chamber under the condition of 20 °C and a natural light/dark cycle. One-week-old seedlings were harvested for DNA and RNA isolation. Genomic DNA was extracted, as described previously [43]. Total RNA was isolated from the seedlings using the E.Z.N.A.^®^ Plant RNA Kit (Omega, Norcross, GA, USA) and then treated with HiScript^TM^ Q RT SuperMix (+gDNA wiper) (Vazyme, Nanjing, China) to remove genomic DNA and to synthesize cDNAs, according to the manufacturer’s instructions.

The extracted DNA and obtained cDNAs were used as PCR templates for the validation of specific RNA editing sites, namely 18 sites of six genes, selected randomly from the prediction, as well as six sites in six genes identified by RNA-seq read mapping. In total, 11 specific primers (Table S2) were designed according to the candidate regions (Table S3). The PCR reaction (20 μL) contained 10 μM of each primer, 1 × EsTaq Mix (Cwbiotech, Beijing, China) and up to 1000 ng of template. The reaction proceeded through 35 cycles after an initial denaturation for 2 min at 94 °C, and was followed by a final extension for 5 min at 72 °C. One thermo cycle was 94 °C for 30 s, annealing for 30 s, and 72 °C for extension. The annealing temperature and extension time were adjusted to the Tm value of the primer pair and the length of product, respectively. The resulting products were separated by 1% agarose gel electrophoresis and were purified with a Universal DNA Purification Kit (Tiangen, Beijing, China), following the manufacturer’s instructions. The homogeneous products of *petL*, *ndhK*, *rpoA*, *rps3*, *ndhD*, *ndhG*, and *rpoB* were sequenced directly from both directions by Sangon Biotech Corporation (Shanghai, China). The products of *ndhB*, *atpA*, *matK*, *ndhA*, and *rpoB* were subsequently ligated into pMD18-T Vector (Takara, Shiga, Japan) and transformed into *Escherichia coli* DH5α. Two positive clones were selected for each fragment to sequence (both directions for *ndhB* and only one direction for others) by Sangon Biotech Corporation. Both DNA and cDNA sequences were spliced using the ContigExpress tool implemented in Vector NTI suite 6.0 (Invitrogen, Waltham, MA, USA), and the RNA editing sites were validated by aligning cDNAs with corresponding genomic DNA sequences using the ClustalW program (http://www.ebi.ac.uk/Tools/msa/clustalw2/) [44].

## 3. Results and Discussion

### 3.1. Prediction of RNA Editing Sites

In total, 34 editing sites present in 15 chloroplast protein-coding genes (Table 2) were predicted by the PREP-Cp program in *Ae. tauschii*, all of which were C-to-U conversions. Among them, *ndh* genes showed the most abundant editing sites with a total of 15, followed by *rpo* genes with a value of 10, and *ycf* with two editing events. Furthermore, 30 out of 34 (88.24%) editing events were observed in the second position of the codon and the rest were in the first position.

### 3.2. Editing Site Detection Using Read Mapping and SNP Calling

A total of 60 sites were identified as RNA editing sites by read mapping and SNP calling, 24 of which agreed with the prediction (Table 3). Among them, only 11 sites were found to be completely edited, and the remaining sites were partially edited, accounting for 81% (49 of 60), which was lower than the 100% value of *A. thaliana* [45]. The abundant, partially edited transcripts, in which not all of the C targets had been converted to Us, might be an intermediate phase in the editing process [15] and might result in more than one protein product encoded by the same gene, suggesting that chloroplast RNA editing was a post-transcriptional process [4]. Furthermore, 20 sites were found to be localized in the intergenic region, presumably affecting 5′ or 3′ UTRs, and two were located within the intron of *rps16*.

**Figure 1:**
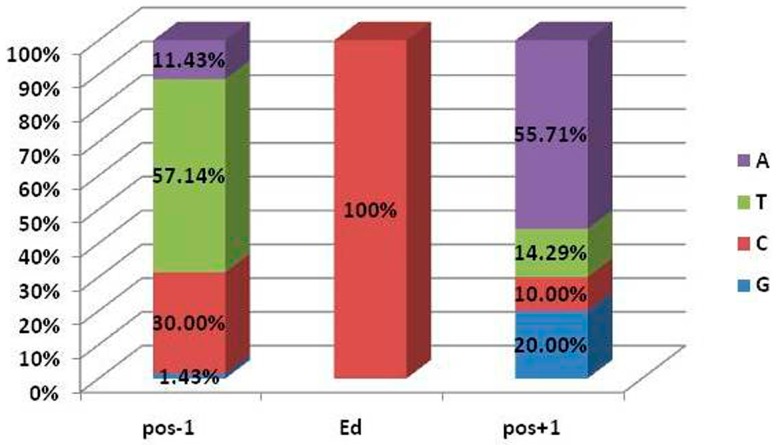
Nucleotide context of editing sites in *Ae. tauschii* (AL8/78). pos −1 and pos +1 indicate the direct upstream and downstream nucleotide of the edited sites (Ed).

**Figure 2:**
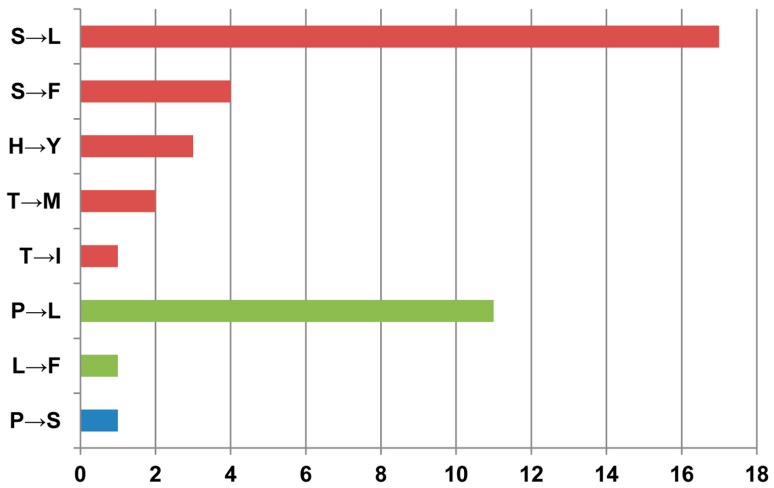
Types and quantities of amino acid exchanges arising from chloroplast RNA editing in *Ae. tauschii* (AL8/78). The red bars represent a conversion of amino acid from hydrophilic to hydrophobic, while blue is for the opposite direction and green is for no hydrophobicity exchanges.

It has been demonstrated that the editing sites in the chloroplasts of seed plants preferred 5′-T–A-3′ flanking bases and/or a 5′-pyrimidine due to the genomic context with lower substitution rates [46,47,48]. Thus, we further analyzed the flanking bases of all 70 editing sites identified via the bioinformatics prediction and RNA-Seq data. Results revealed that 31.43% (22 of 70) of sites showed a tCa bias and 81.43% (57 of 70) possessed tCn or nCa trinucleotides (Figure 1). Additionally, a 5′-pyrimidine was observed in 61 sites, accounting for 87.14%, of which thymine accounted for 57.14% and cytosine accounted for 30.00%. Furthermore, all the editing events in the protein-coding genes could result in eight types of amino acid transitions (Figure 2). Among them, 27 out of 40 editing events (67.5%) led to a conversion from hydrophilic amino acid to hydrophobic amino acid (Figure 2, red bar), with only one changing from hydrophobic amino acid to hydrophilic amino acid (P→S) (Figure 2, blue bar), and the remaining 12 without hydrophobicity exchanges (Figure 2, green bar). Moreover, most of the amino acid conversions affected by RNA editing were serine to leucine (17), followed by proline to leucine (11). This was most likely due to serine being encoded by tCn and proline by the cCn codon. The low re-mutation biased genomic context could result in the conservative (functionally similar) amino acid exchange, probably without negative effects on protein function [49].

Additionally, six silent editing sites with no impact on the encoded amino acid were also detected, including pistil-specific *atpA*-234 and *rpl16*-36, spike-specific *psbB*-414, and *rps3*-30 edited in all tissues. Editing events at sites *rps18*-448 and *ndhF*-1891 generated stop codons, resulting in the premature termination of transcription, which only occurred in pistils with relatively low efficiency (9%). Furthermore, editing events at *ndhB*-586, *ndhB*-611, *ndhB*-704, and *ndhB*-1481 on the sense-strand, as well as *ndhB*-836, *ndhB*-149, *ndhA*-1070, and *ndhA*-563 on the antisense-strand, were found to only occur in leaves, while *rps4*-305 was specifically edited in seeds.

### 3.3. Validation of the RNA Editing Sites by RT-PCR Analysis

To validate the predicted RNA editing sites, 24 sites of 11 genes were randomly selected for PCR and RT-PCR analyses. By comparing cDNAs with genomic sequences, 23 sites in 11 genes were proven to be true (approximately 95.83%). Only *rpoB*-617 was not validated, as nucleotide C was present in both the mRNA and genomic DNA (Figure S1). In addition to false positives resulting from the bioinformatics prediction, one possible explanation was that *rpoB*-617 was edited in a tissue- or stage-specific manner, or modulated under different environmental conditions, which were not included in current study.

Generally, there are three methods to identify RNA editing sites, namely bioinformatics prediction and RNA-Seq, as well as molecular cloning. Molecular cloning is a common, widely used and accurate method to identify RNA editing in different species. Kugita et al. [50] verified a total of 509 C-to-U and 433 U-to-C conversions in hornwort chloroplasts and showed that RNA editing in hornwort chloroplasts made more than half the genes function; Jiang et al. [51] identified 54 editing sites in the cotton chloroplast genome, which was the highest editing frequency reported in angiosperms. However, this method requires a series of time-consuming and tedious experimental procedures, making it not suitable for genome-wide identification. In light of the evolutionary conservation, bioinformatics prediction tools have been developed to identify RNA editing, such as PREP-Cp and CURE (Cytidine to Uridine Recognizing Editor) [52]. In the cp genome of *Cycas taitungensis*, 85 editing sites were found in 25 transcripts using a combination of prediction by CURE and experimental determination [53]. In coconut cpDNA, RT-PCR analysis confirmed editing at 64 of the 83 PREP-Cp predicted RNA editing sites out of 27 genes [54]. With the advent of high-throughput sequencing, RNA-Seq technology provides an opportunity to investigate RNA editing sites at the transcriptome level. By read mapping and SNP calling, SNPs that emerged repeatedly and existed stable could be interpreted as edited sites. It has been gradually used to identify RNA editing on a large scale [2,55]. However, edited targets can be easily neglected, or SNPs can be falsely interpreted as true edited targets using this method [55]. Thus, to efficiently and accurately identify RNA editing sites in *Aegilops tauschii* L. chloroplasts, we performed identification through a combination of these three methods, which provide a systematic and comprehensive RNA editing profile of the transcriptome of *Aegilops tauschii* L. chloroplasts.

### 3.4. Impact of RNA Editing on Protein Structure

To preliminarily understand the biological functions of RNA editing, the secondary structures of these proteins, encoded by 15 genes with editing events, were analyzed using CFSSP and Phobius tools. Results showed that *petB*, *rpoA*, and *rps8* possessed the same secondary structures, before and after editing, while editing events of the remaining 12 genes could alter the composition of the secondary structures around the editing sites (Table S4). More than half of the edited genes preferred to form new a α-helix and β-sheet, and also more than half of them underwent a decrease of turn structures at up- and down-stream regions around the editing codon (Table S5).

**Figure 3:**
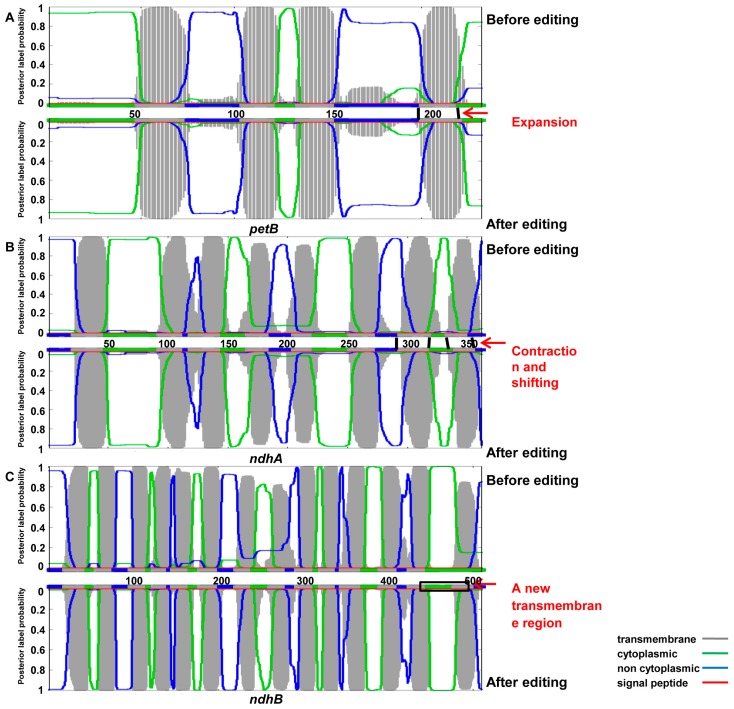
Transmembrane topology of *petB*, *ndhA* and *ndhB* before and after editing. (**A**) The transmembrane region of *petB* expanded to the C terminal of the protein after editing; (**B**) The transmembrane region of *ndhA* displayed a contraction and shift after editing; (**C**) *NdhB* showed a larger transmembrane region after editing.

In the cp genome of *Chlamydomonas reinhardtii*, no editing was observed at position 204 of the *petB* gene encoding a cytochrome b_6_ subunit after introducing a proline residue in place of a leucine residue [56]. This gene modification led to a defective assembly of cytochrome b_6_f complexes, consistent with a block in the photosynthetic electron transfer so that the transformants displayed non-phototrophic phenotypes. The 204 position of *petB* in *C. reinhardtii* corresponded to the *petB*-662 in *Ae. tauschii* (AL8/78), which was in the proline codon CCA at the DNA level and was subsequently edited to the leucine codon CTA at the RNA level. Although the editing event at *petB*-662 had no impact on the hydrophobicity of the amino acids, the composition of the secondary structures or the number of transmembrane structures, the transmembrane region at codons 201 to 220 expanded to the C terminal of the protein (Figure 3A). We thus speculated that the editing event at *petB*-662 contributed to maintaining the functional activity of cytochrome b_6_f in *Ae. tauschii*.

There was no change in the number of transmembrane structures of *ndhA*, before and after editing, which encoded NADH dehydrogenase subunit A. Nevertheless, a contraction in the region of codons 296 to 323 was observed and the transmembrane region at codons 335 to 353 shifted to the C terminal (Figure 3B). The edited codons also restored universally conserved amino acids in the *ndhA*-encoded peptides of other chloroplast species, as was found in maize [57]. In addition, *ndhB*, with all sites edited, revealed a new transmembrane region at codons 449 to 510 (Figure 3C). *NdhB* editing site III was reported to remain unedited in the non-photosynthetic mutants of tobacco, as well as during the etiolated stage of the seedling development of maize, whereas it was fully edited in plants with an intact photosynthetic apparatus, indicating that the editing events in *ndhB* of *Ae. tauschii* might occur with a similar developmental stage-dependent manner, suggesting an unexpected link between *ndhB* editing and photosynthesis and light-induced chloroplast development [58].

### 3.5. Comparative Analysis of Chloroplast RNA Editing Sites among Poaceae Species

A total of 31 conserved RNA editing sites in 15 chloroplast protein-coding genes were systematically compared in *Ae. tauschii*, *H. vulgare*, *L. perenne*, *O. sativa*, *S. officinarum*, and *Z. mays*, 14 sites of which were found to be fully or partially edited (Table 4), establishing an evolutionarily conserved feature among these six species. No editing events occurred at *ndhA*-1, *ndhK*-1, *rpl2*-1 and *rps14*-1, and the absence of edited *rpl2*-1 was specific to *Ae. tauschii*. *NdhK*-1 was not edited in *Ae. tauschii*, and nucleotide C was present at the genomic DNA level. For the other three sites, nucleotide T already existed at the genomic DNA level. These sites had probably re-mutated to T at the DNA level to remedy detrimental genomic mutations that occurred after the water-to-land transition of land plants [59]. In terms of *ndhB*-1, *ndhB*-5 and *ndhB*-8, *Ae. tauschii*, *H. vulgare*, *L. perenne* and *O. sativa* shared edited *ndhB*-5 and *ndhB*-8, whereas *ndhB*-1 was only edited in *Triticeae* crops, suggesting that patterns of RNA editing were in accordance with the phylogenetic relationship (Figure S2) and there was a specific evolutionary force to drive the RNA editing events in these species.

**Figure 4:**
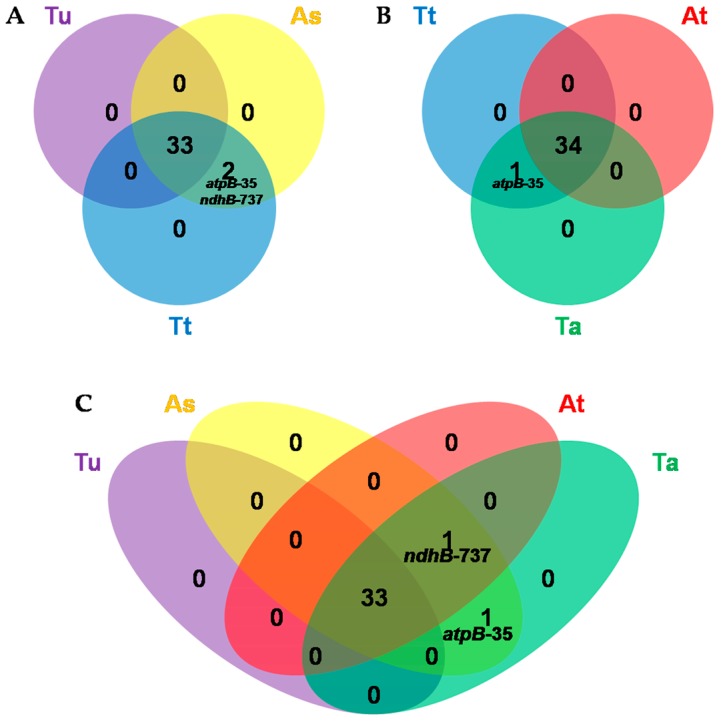
Comparison of RNA editing sites in chloroplast genes of *T. aestivum* and its genome donors. (**A**) Comparison among *T. urartu*, *Ae. speltoides* and *T. turgidum*; (**B**) Comparison among *T. turgidum*, *Ae. tauschii* and *T. aestivum*; (**C**) Comparison among *T. urartu*, *Ae. speltoides*, *Ae. tauschii* and *T. aestivum*. Tu indicates *T. urartu* (PI428335); As indicates *Ae. speltoides* (AE918, TA1796 and PI487232); At indicates *Ae. tauschii* (AL8/78); Tt indicates *T. turgidum* ssp. *dicoccoides* (TA0073, TA0060 and TA1133); and Ta indicates *T. aestivum* cv CS (TA3008). *T. urartu*, *Triticum urartu* L. *Ae. speltoides*, *Aegilops speltoides* L. *T. turgidum, Triticum turgidum* L. *Ae. tauschii*, *Aegilops tauschii* L. *T. aestivum*, *Triticum aestivum* L.

Furthermore, to trace the evolution information of polyploid wheat from the chloroplast RNA editing point of view, we investigated and compared the chloroplast RNA editing patterns between *Ae. tauschii* and other wheat relatives including *T. urartu*, *Ae. speltoides* and *T. turgidum*. In total, 35 editing sites were detected in *Ae. speltoides*, *T. turgidum*, and *T. aestivum*, while only 33 sites were found in *T. urartu* (Tables S6–S9). Moreover, common wheat and its genome donors shared the 33 sites in 15 protein-coding genes of *T. urartu* (Figure 4), indicating that a highly conserved characteristic of RNA editing was present during the evolution of common wheat. It is worth noting that *atpB*-35 and *ndhB*-737 were edited in *Ae. speltoides* and *T. turgidum* compared to *T. urartu* (Figure 4A). As *atpB*-35 and *ndhB*-737 in *T. urartu*, as well as *atpB*-35 in *Ae. tauschii*, were not edited, the encoded T in the DNA level might be a result of re-mutation to repair the deficient transcripts that arose from mutations as described previously. If we posited that editing sites remained stable when the genome progenitors evolved to common wheat, undergoing the natural cross and chromosome doubling, *atpB*-35 and *ndhB*-737 of *T. turgidum* might come from *Ae. speltoides*. As predicted, the editing event at *atpB*-35 was not observed in *Ae. tauschii* but occurred in *T. aestivum* (Figure 4B,C), suggesting that *atpB*-35 of *T. aestivum* came from *T. turgidum* and originated from *Ae. speltoides*. However, the editing event at *ndhB*-737 also occurred in *Ae. tauschii*. Thus, further study is needed to reveal the origin of *ndhB*-737 in common wheat.

## 4. Conclusions

Here, we identified the RNA editing sites in the *Ae. tauschii* chloroplast genome through bioinformatics prediction, combined with RNA-Seq mapping and molecular cloning. A total of 34 editing sites in 15 protein-coding genes were predicted by PREP-Cp, of which 23 sites in 12 genes were validated by RNA-Seq mapping and 17 sites in six genes by molecular cloning. Additionally, RNA-Seq mapping detected 60 sites, seven of which were confirmed experimentally. *Ae. tauschii* shared similar patterns and features of RNA editing with other land plants and editing events could result in the alteration of the secondary structures of the encoded protein, which might have an impact on biological functions. Finally, comparative analysis was performed to identify the evolutionarily conserved or species-specific sites. Two sites, including *atpB*-35 and *ndhB*-737, were found to underlie some origin and evolutionary information of common wheat. This study, for the first time, reported on RNA editing in *Aegilops tauschii* L. and compared the RNA editing pattern among wheat relative species, which not only shed light on the evolution of wheat from the point of view of RNA editing, but also lay the foundation for further studies to identify the mechanisms of RNA editing in wheat and beyond.

## Figures and Tables

**Table 1:** Information of complete chloroplast genome for comparative analysis of RNA editing sites in protein-coding genes among Poaceae species.

Material Name	GenBank Accession
*Ae. tauschii* (AL8/78)	KJ614412.1
*T. urartu* (PI428335)	KJ614411.1
*Ae. speltoides* ssp. *ligustica* (AE918)	KJ614404.1
*Ae. speltoides* ssp. *ligustica* (TA1796)	KJ614405.1
*Ae. speltoides* ssp. *speltoides* (PI487232)	KJ614406.1
*T. turgidum* ssp. *dicoccoides* (TA0073)	KJ614400.1
*T. turgidum* ssp. *dicoccoides* (TA0060)	KJ614401.1
*T. turgidum* ssp. *dicoccoides* (TA1133)	KJ614402.1
*T. aestivum* ssp. *aestivum* cv Chinese Spring (CS) (TA3008)	KJ614396.1

**Table 2:** RNA editing sites in chloroplast protein-coding genes of *Ae. tauschii* (AL8/78) predicted by PREP-Cp.

Gene	Nucleotide Position	Amino Acid Position	Codon Conversion	Amino Acid Conversion
*ndhA*	473 ^a^	158	tCa>tTa	S>L
	563 ^a^	188	tCa>tTa	S>L
	1070	357	tCt>tTt	S>F
*atpA*	1148 ^a^	383	tCa>tTa	S>L
*atpB*	1487	496	tCg>tTg	S>L
*matK*	1261 ^a^	421	Cat>Tat	H>Y
*ndhB*	149^a^	50	tCa>tTa	S>L
	467 ^a^	156	cCa>cTa	P>L
	586 ^a^	196	Cat>Tat	H>Y
	611 ^a^	204	tCa>tTa	S>L
	704 ^a^	235	tCc>tTc	S>F
	737 ^a^	246	cCa>cTa	P>L
	830 ^a^	277	tCa>tTa	S>L
	836 ^a^	279	tCa>tTa	S>L
	1481 ^a^	494	cCa>cTa	P>L
*ndhD*	878 ^a^	293	tCa>tTa	S>L
*ndhF*	62	21	tCa>tTa	S>L
	1487	496	aCg>aTg	T>M
*petB*	662	221	cCa>cTa	P>L
*rpoC2*	2009	670	cCa>cTa	P>L
	2030	677	cCa>cTa	P>L
	2158	720	Ccc>Tcc	P>S
	3002	1001	cCg>cTg	P>L
	4031	1344	tCg>tTg	S>L
*rpl2*	62	21	aCt>aTt	T>I
*rpl20*	308	103	tCa>tTa	S>L
*rpoA*	1009	337	Ctc>Ttc	L>F
*rpoB*	467 ^a^	156	tCg>tTg	S>L
	545 ^a^	182	tCa>tTa	S>L
	560 ^a^	187	tCg>tTg	S>L
	617 ^b^	206	cCg>cTg	P>L
*rps8*	182	61	tCa>tTa	S>L
*ycf3*	44	15	tCc>tTc	S>F
	191	64	aCg>aTg	T>M

Capitals in column Codon Conversion indicate target nucleotides. ^a^ Editing sites with experimental verification. ^b^ Unedited by experimental verification.

**Table 3:** Cytidine (C) to uridine (U) editing sites in chloroplast genome of *Ae. tauschii* (AL8/78) validated by RNA-Seq data.

**Genome Position**	**Gene Position**	**Codon**	**Amino Acid**	**Leaf**					**Pistil**	**Root**				**Seed**			**Sheath**				**Spike**			**Stamen**	**Stem**		
				**1 ^b^**	**2**	**3**	**4**	**5**	**1**	**1**	**2**	**3**	**4**	**1**	**2**	**3**	**1**	**2**	**3**	**4**	**1**	**2**	**3**	**1**	**1**	**2**	**3**
Sense-strand																											
3090	~*psbK*								22																		
3551	~*psbK*								7																		
8259	*psbI*~*psbD*			35 ^c^	36	37	36	37				47	44				33	29	32	33			25	22			
8481	*psbI*~*psbD*								9																		
11,311	*psbC*~*psbZ*			21		19	20		18										10				10		9		
20,093	*rpoB*-467 ^a^	tCg	S>L					38	28								66	75	78	90				72			32
20,171	*rpoB*-545 ^a^	tCa	S>L						48								70	86	88	78				71			
20,186	*rpoB*-560 ^a^	tCg	S>L						57								73	86	80	85				69			
29,181	*rpoC2*-4031 ^a^	tCg	S>L	94	+	+	91	98	+			+	82	71	60	73	97	99	93	97	92		+	92	99		98
32,404	*atpH*~*atpF*						18	18						15													
34,440	*atpA*-234	ggC	G>G						11																		
35,354	*atpA*-1148 ^a^	tCa	S>L	99	98	99	99	99		83	94	98	91	94	91	87	96	90	94	96	95		94	71	93	95	94
42,415	*atpA*~*trnS-GGA*																	14									
57,960	*ycf4*~*cemA*			52	50	67	48	55	12								37	42	27	31				23			26
62,758	*petL*-56 ^d^	cCa	P>L	83	75	62	77	60									58		36	30					83		
65,352	*rps18*-448	Cga	R>Stop						9																		
68,447	*psbB*-414	atC	L>L																				17				
71,835	*petB*-662 ^a^	cCa	P>L	96	98	97	98	90						43		27				34			66	58	26		13
127,814	*ndhB*-149 ^a^	tCa	S>L	94																96							
128,251	*ndhB*-586 ^a^	Cat	H>Y	95	88	91	93	93																			
128,276	*ndhB*-611 ^a^	tCa	S>L	95		93	95	94																			
**Genome Position**	**Gene Position**	**Codon**	**Amino Acid**	**Leaf**					**Pistil**	**Root**				**Seed**			**Sheath**				**Spike**			**Stamen**	**Stem**		
				**1**	**2**	**3**	**4**	**5**	**1**	**1**	**2**	**3**	**4**	**1**	**2**	**3**	**1**	**2**	**3**	**4**	**1**	**2**	**3**	**1**	**1**	**2**	**3**
Sense-strand																											
128,369	*ndhB*-704 ^a^	tCc	S>F				92																				
129,858	*ndhB*-1481 ^a^	cCa	P>L	96	99	99		97																			
Antisense-strand																											
1956	*matK*-1261 ^a^	Cat	H>Y	66	45	41	69	52	13			36					69	55	38	69	55		50	36	61		71
5100	*rps16*-intron			19	17	22	23	22	89	54	51	50		65			36	33	32	37	83		83	60	66	70	60
5179	*rps16*-intron									15	15	23															
36,673	*rps14*~psaB			45	45	45	44	45									20		20	14			19	23			8
42,982	*ycf3*-191 ^a^	aCg	T>M	79	83	82	81	80	63	44	55	45	39			49	70	69	67	63	64		74	64	72		76
43,880	*ycf3*-44 ^a^	tCc	S>F	92	92	96	97	92	40	71	68	70	26	46			70	70	69	82	42		50	43	69	77	74
44,727	*ycf3*~rps4			78	68	71	75	65	38					41			35	29	35	19					20	21	
44,868	*ycf3*~rps4								11																		
45,244	*rps4*-305	tCa	S>L											20													
49,393	*ndhK*-125 ^d^	cCa	P>L	99	98	99	99	99	+			70	+	71			96	97	94	94	+		93	89	95		89
49,858	*ndhC*-13	Cac	H>Y	98	98	98	99	99	75	86	83	63	63	86			86	92	85	91			56	80	83		63
61,027	*psbL*-111	ttC	F>F	77	83	83	81	49	76								47	45	33	29				56			
65,383	*trnP-GGG*~*rpl20*					24																					
65,632	*rpl20*-308 ^a^	tCa	S>L			11		24									60	38	63	75				56			
67,968	*clpP*~*psbN*			31																							
73,402	*psbN*~*rpoA*			45	50	51	51	45				35					19	12	13	21	20			15	14		22
73,980	*rpoA*-527 ^d^	tCc	S>F	30	34	30	28	28		23	18	31	35	79	64	72	81	84	81	90	84	75	85	41	87	82	86
74,553	*rpoA*~rps11			41	44	44	45	41	21		19	11	29	15	12	22	21	20	22	18	18		18	17	23	20	20
76,074	*rps8*-182 ^a^	tCa	S>L	89	90	89	90	90	96	83	86	83	84	96	96	95	97	97	97	97	93	95	93	96	95	95	95
77,285	*rpl16*-36	ccC	P>P						18																		
78,313	*rpl16*~*rps3*								92																		
**Genome Position**	**Gene Position**	**Codon**	**Amino Acid**	**Leaf**					**Pistil**	**Root**				**Seed**			**Sheath**				**Spike**			**Stamen**	**Stem**		
				**1**	**2**	**3**	**4**	**5**	**1**	**1**	**2**	**3**	**4**	**1**	**2**	**3**	**1**	**2**	**3**	**4**	**1**	**2**	**3**	**1**	**1**	**2**	**3**
Antisense-strand																											
79,174	*rps3*-30 ^d^	ttC	F>F	62	57	62	66	59	77	58	66	71	69	78	82	63	66	68	69	71	68	63	63	62	56	57	48
81,816	*rpl2*~*rpl23*								82								87	88	88								
86,095	*ndhB*-836 ^a^	tCa	S>L					99																			
87,176	*ndhB*-467 ^a^	cCa	P>L	99	95	96	97	98										96		89							
87,494	*ndhB*-149 ^a^	tCa	S>L		95																						
96,705	*rps7*~*trnN-GUU*			6			12																				
101,658	*ndhF*-1891	Cag	Q>Stop						9																		
103,487	*ndhF*-62 ^a^	tCa	S>L	45	43	44	49	51						28			39	39	43	18			21	17	44		37
106,742	*ndhD*-1398 ^d^	atC	L>L	36	28	37	34	37																			23
107,262	*ndhD*-878 ^a^^,d^	tCa	S>L	99	98	99	99	98				+		89	95		89	81	89	77	84		77	94	89	+	83
109,393	*ndhE*~*ndhG*							32																			
109,684	*ndhG*-347 ^d^	cCa	P>L	98	97	97	98	96									65	59	64	72				40	78		65
110,034	*ndhG*~*ndhI*			38	27	22	28	19																			
110,946	*ndhA*-1070 ^a^	tCt	S>F	75			64	92																			
111,453	*ndhA*-563 ^a^	tCa	S>L	13																							
112,572	*ndhA*-473 ^a^	tCa	S>L			+											45	33									
		No. of site		35	29	33	33	35	27	9	10	16	11	16	7	8	28	28	28	29	13	3	19	25	20	9	21

Capitals in column Codon indicate target nucleotides. ^a^ Editing sites consistent with the prediction. ^b^ Biological replicates of each tissue. ^c^ This value indicates editing efficiency (%). ^d^ Editing sites validated by direct sequencing of PCR products. +, Complete editing (editing efficiency, 100%). Blank, no editing/not yet determined.

**Table 4:** Comparative analysis of RNA editing sites in chloroplast genes among six Poaceae species.

Gene	Site	Codon Position	Edited Codon	Amino Acid Conversion	*Ae. tauschii*	*H. vulgare*	*L. perenne*	*O. sativa*	*S. officinarum*	*Z. mays*
*matK*	1	420 (421)	Cat	H→Y	+	+	+ ^a^	+		
*rpoB*	1	156	tCa	S→L	+	+	+ ^a^	+ ^a^	+	+
	2	182	tCa	S→L	+	+	+ ^a^	+ ^a^	+	+
	3	187	tCg	S→L	+	+	+ ^a^	+ ^a^ uCa	+	+
	4	206	cCg	P→L	+	−	−	−	−	+
*rps14*	1	27	tCa	S→L	(−)		(−)	+	+ ^a^	+
*atpA*	1	383	tCa	S→L	+		+	+	+	+
*ycf3*	1	15	tCc	S→F	+		+	(−)	−	+
	2	62 (64)	aCg	T→M	+		+	+ ^a^	+	+ ^a^
*ndhG*	1	116	cCa	P→L	+ ^a^		+	+		(−)
*rpl20*	1	103	tCa	S→L	+		+ ^a^	(−)	+^a^	+
*psbL*	1	37	ttC	F→F	+ ^a^		+ ^a^	(−)		
*rps8*	1	61	tCa	S→L	+		+	+	+	+
*rpl2*	1	1	aCg	T→M	(−)	+	+ ^a^	+ ^a^	+^a^	+
*ndhB*	1	50	tCa	S→L	+	+	+	(−)	(−)	(−)
	2	156	cCa	P→L	+	+	+	+	+	+
	3	196	Cat	H→Y	+	+	+	+	+	+
	4	204	tCa	S→L	+	+	+	+ ^a^	+	+
	5	235	tCc	S→F	+	+	+	+	(−)	(−)
	6	246	cCa	P→L	+	+	+	+	+	+
	7	277	tCa	S→L	+	+	+	+	+	+
	8	279	tCa	S→L	+	+	+	+	(−)	(−)
	9	494	cCa	P→L	+	+	+	+ ^a^	+	+
*ndhF*	1	21	tCa	S→L	+		+	+	+ ^a^	+
*ndhD*	1	295 (293)	tCa	S→L	+	+	+ ^a^	+	+	+
*ndhA*	1	17	tCa	S→L	(−)	+	+	(−)	+	+
	2	158	tCa	S→L	+	+	+	+	+	+
	3	188	tCa	S→L	+	+	+	+	+	+
	4	357	tCc	S→F	+	+	(−)	+	+	+
*ndhK*	1	2	gtC	V→V	−		+			
	2	43(42)	cCa	P→L	+		+			

Capitals in column Edited Codon indicate target nucleotides. “+”, complete editing; “−”, no editing and C encoded in DNA; “(−)”, no editing and T encoded in DNA; “+ ^a^”, partial editing; Blank, no information available/not yet determined. *Ae. tauschii*, *Aegilops tauschii* L. *H. vulgare*, *Hordeum vulgare* L. *L. perenne*, *Lolium perenne* L. *O. sativa*, *Oryza sativa* L. *S. officinarum*, *Saccharum officinarum* L. *Z. mays*, *Zea mays* L.

## Supplementary Materials

The following are available online at www.mdpi.com/2073-4425/8/1/13/s1, Figure S1: RNA editing sites confirmed experimentally, except *rpoB*-617 which was not edited. Arrow points to the editing site; Figure S2: The phylogeny of the compared six Poaceae species, *Ae. tauschii*, *H. vulgare*, *L. perenne*, *O. sativa*, *S. officinarum* and *Z. mays*. The phylogenetic tree of the compared six Poaceae species was reconstructed using maximum likelihood (ML) based on the RNA editing sites’ composition; Table S1: Information of RNA-Seq data of *Ae. tauschii* (AL8/78); Table S2: Primer information used in this study; Table S3: RNA editing sites validated by each primer pair; Table S4: Quantity of protein secondary structures before and after editing; Table S5: Conversion of protein secondary structure before and after editing; Table S6: RNA editing sites in chloroplast genes of *T. aestivum* cv CS (TA3008) predicted by PREP-Cp; Table S7: RNA editing sites in chloroplast genes of *T. urartu* (PI428335) predicted by PREP-Cp; Table S8: RNA editing sites in chloroplast genes of *Ae. speltoides* (AE918, TA1796 and PI487232) predicted by PREP-Cp; Table S9: RNA editing sites in chloroplast genes of *T. turgidum* ssp. *dicoccoides* (TA0073, TA0060 and TA1133) predicted by PREP-Cp.
