# Supplementary material for: Identification and Analysis of RNA Editing Sites in the Chloroplast Transcripts of Aegilops tauschii L
Source: Genes (Basel). 2016 Dec 30;8(1):13. doi: 10.3390/genes8010013 (PMC5295008; doi:10.3390/genes8010013)
Supplement: Supplementary file 1 [file genes-08-00013-s001.pdf]

Genes **2017**, *8*, 13; doi:10.3390/genes8010013

Supplementary Materials: Identification and Analysis of RNA Editing Sites in the Chloroplast Transcripts of *Aegilops tauschii* L.

Mengxing Wang, Hui Liu, Lingqiao Ge, Guangwei Xing, Meng Wang, Song Weining and Xiaojun Nie

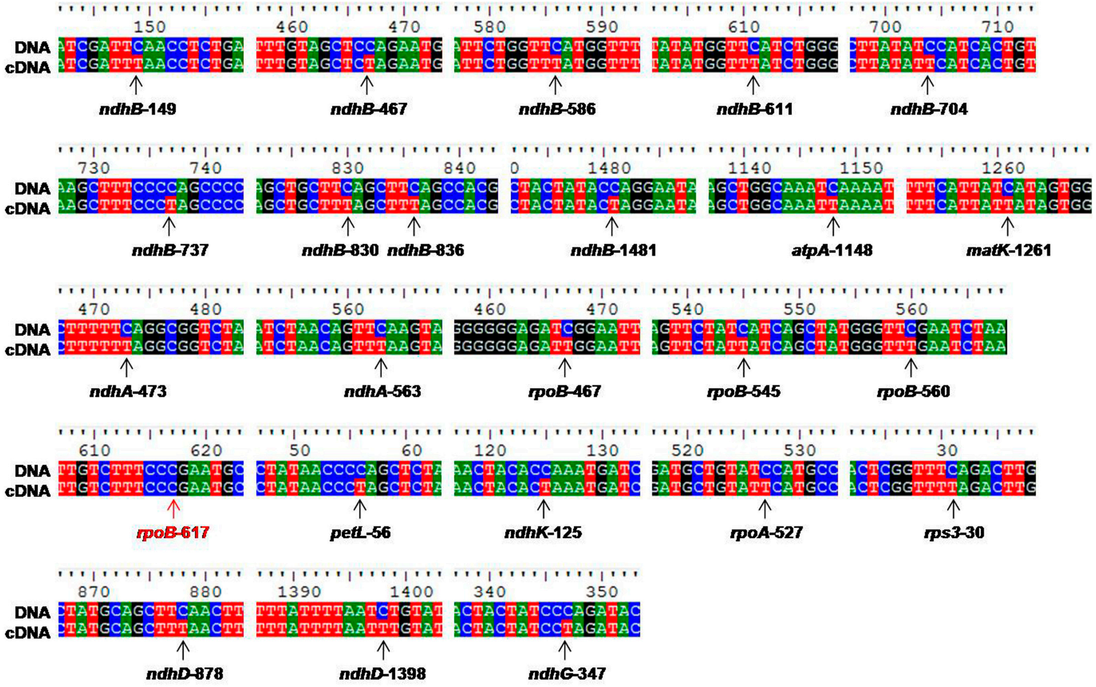

Figure S1. RNA editing sites confirmed experimentally, except *rpoB*-617 which was not edited. Arrow points to the editing site.

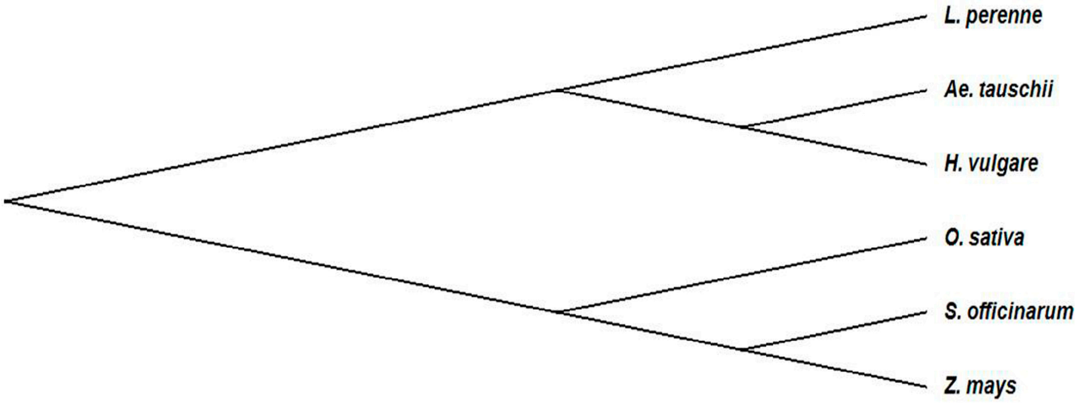

Figure S2. The phylogeny of the compared six Poaceae species, *Ae. tauschii*, *H. vulgare*, *L. perenne*, *O. sativa*, *S. officinarum* and *Z. mays*. The phylogenetic tree of the compared six Poaceae species was reconstructed using maximum likelihood (ML) based on the RNA editing sites' composition.

Table S1. Information of RNA-Seq data of *A. tauschii* (AL8/78).

Tissue	SRA Accession	Tissue	SRA Accession
Leaf1	SRX209402	Seed3	SRX209414
Leaf2	SRX209403	Sheath1	SRX209415
Leaf3	SRX209404	Sheath2	SRX209416
Leaf4	SRX209405	Sheath3	SRX209417
Leaf5	SRX209406	Sheath4	SRX209418
Pistil1	SRX209407	Spike1	SRX209419
Root1	SRX209408	Spike2	SRX209420
Root2	SRX209409	Spike3	SRX209421
Root3	SRX209410	Stamen1	SRX209422
Root4	SRX209411	Stem1	SRX209423
Seed1	SRX209412, ,	Stem2	SRX209424
Seed2	SRX209413	Stem3	SRX209425

Table S2. Primer information.

Name	Sequence (5′ to 3′)	Length (bp)	Tm (°C)	GC%	Product Length (bp)
ndhB-F	GAAAGCGTTTCATTTGCTTCTC	22	56.3	40.9	1477
ndhB-R	CTAAAAGAGGGTATCCTGAGCAAT	24	58.6	41.7	
atpA-F	CTGCGGATCTATTCAATGC	19	55.4	47.4	153
atpA-R	CAAAGGCTTGTAACTCTGCGA	21	58.0	47.6	
matK-F	GAATCAAATGCTGGAGAAGTCA	22	56.3	40.9	338
matK-R	CCGAACCCAATCGTTGC	17	57.0	58.8	
ndhA-F	ATTGGTCTTCTTATGGCAGG	20	55.8	45.0	288
ndhA-R	TTCCTCCGCTTCTGGTAAAT	20	55.8	45.0	
rpoB-F	GGGAATTTCTATCTACACTGGCA	23	58.4	43.5	367
rpoB-R	TCGACCAATCCTTCCTAATTCAC	23	58.4	43.5	
petL-F	AACTAGTTATTTTGGTTTTCTACTG	25	51.4	28.0	85
petL-R	TCAAATAAGGCGTATCTTGT	20	49.2	35.0	
ndhK-F	AAGGAAAAGATTCTATTGAGACAGT	25	53.0	32.0	312
ndhK-R	TTTGCTCGTATAATCTCACTAAAGA	25	53.0	32.0	
rpoA-F	GCCTTTATTATGGTCGTTTCA	21	51.7	38.1	510
rpoA-R	GTTTTTCATTTCCATTCCCAC	21	51.7	38.1	
rps3-F	GGACAAAAAATAAATCCACTCGGT	24	54.4	37.5	433
rps3-R	TAGGCTGTCTATAAGGTTCTTTCA	24	54.4	37.5	
ndhD-F	TCGGAGTTCTGGGTATGG	18	54.9	55.6	932
ndhD-R	TACCCTGTCAACGGATAGGGAG	22	59.5	54.5	
ndhG-F	GTAATGTTCGTAAATGGCTCAG	22	54.0	40.9	249
ndhG-R	GGAAATGAGTTCAAACGGAAGA	22	54.0	40.9	

Table S3. RNA editing sites validated by each primer pair.

Primer Pair	RNA Editing Sites to Be Validated
ndhB-F/ndhB-R	All the 9 sites in *ndhB*
atpA-F/atpA-R	*atpA*-1148
matK-F/matK-R	*matK*-1261
ndhA-F/ndhA-R	*ndhA*-473, *ndhA*-563
rpoB-F/rpoB-R	*rpoB*-467, *rpoB*-545, *rpoB*-560, *rpoB*-617
petL-F/petL-R	*petL*-56
ndhK-F/ndhK-R	*ndhK*-125
rpoA-F/rpoA-R	*rpoA*-527
rps3-F/rps3-R	*rps3*-30
ndhD-F/ndhD-R	*ndhD*-878, *ndhD*-1398
ndhG-F /ndhG-R	*ndhG*-347

Table S4. Quantity of protein secondary structures before and after editing.

Gene		Secondary Structure			Gene		Secondary Structure		
		H	E	T			H	E	T
atpA	B	432	209	71	*rpl2*	B	88	39	25
	A	432	209	70		A	91	39	26
atpB	B	349	212	61	*rpl20*	B	74	83	14
	A	349	212	62		A	75	84	14
matK	B	368	417	50	*rpoA*	B	254	147	47
	A	368	418	49		A	254	147	47
ndhA	B	306	254	23	*rpoB*	B	777	716	157
	A	309	260	21		A	778	729	153
ndhB	B	373	378	45	*rpoC2*	B	1021	945	210
	A	378	399	44		A	1027	955	207
ndhD	B	402	337	30	*rps8*	B	96	69	18
	A	402	344	29		A	96	69	18
ndhF	B	539	528	57	*ycf3*	B	105	75	29
	A	541	528	57		A	109	85	28
petB	B	153	178	20					
	A	153	178	20					

H, E and T indicates α-helix, β-sheet and turn, respectively. B, before editing; A, after editing.

Table S5. Conversion of protein secondary structure before and after editing.

Secondary Structure	Conversion	Gene	Number
α-helix	Increase	*ndhA, ndhB, ndhF, rpl2, rpl20, rpoB, rpoC2, ycf3*	8
	Reduce	*None*	0
	No change	*atpA, atpB, matK, ndhD, petB, rpoA, rps8*	7
β-sheet	Increase	*matK, ndhA, ndhB, ndhD, rpl20, rpoB, rpoC2, ycf3*	8
	Reduce	*None*	0
	No change	*atpA, atpB, ndhF, petB, rpl2, rpoA, rps8*	7
turn	Increase	*atpB, rpl2*	2
	Reduce	*atpA, matK, ndhA, ndhB, ndhD, rpoB, rpoC2, ycf3*	8
	No change	*ndhF, petB, rpl20, rpoA, rps8*	5

Table S6. RNA editing sites in chloroplast genes of *T. aestivum* cv CS (TA3008) predicted by Prep-Cp.

Gene	Nucleotide Position	Amino Acid Position	Codon Conversion	Amino Acid Conversion
ndhA	473	158	tCa→tTa	S→L
	563	188	tCa→tTa	S→L
	1070	357	tCt→tTt	S→F
atpA	1148	383	tCa→tTa	S→L
atpB	35	12	gCt→gTt	A→V
	1487	496	tCg→tTg	S→L
matK	1261	421	Cat→Tat	H→Y
ndhB	149	50	tCa→tTa	S→L
	467	156	cCa→cTa	P→L
	586	196	Cat→Tat	H→Y
	611	204	tCa→tTa	S→L
	704	235	tCc→tTc	S→F
	737	246	cCa→cTa	P→L
	830	277	tCa→tTa	S→L
	836	279	tCa→tTa	S→L

	1481	494	cCa→cTa	P→L
ndhD	878	293	tCa→tTa	S→L
ndhF	62	21	tCa→tTa	S→L
	1487	496	aCg→aTg	T→M
petB	662	221	cCa→cTa	P→L
	2009	670	cCa→cTa	P→L
	2030	677	cCa→cTa	P→L
rpoC2	2158	720	Ccc→Tcc	P→S
	3002	1001	cCg→cTg	P→L
	4031	1344	tCg→tTg	S→L
rpl2	62	21	aCt→aTt	T→I
rpl20	308	103	tCa→tTa	S→L
rpoA	1009	337	Ctc→Ttc	L→F
	467	156	tCg→tTg	S→L
rpoB	545	182	tCa→tTa	S→L
	560	187	tCg→tTg	S→L
	617	206	cCg→cTg	P→L
rps8	182	61	tCa→tTa	S→L
ycf3	44	15	tCc→tTc	S→F
	191	64	aCg→aTg	T→M

Capitals in column Codon Conversion indicate target nucleotides.

Table S7. RNA editing sites in chloroplast genes of *T. urartu* (PI428335) predicted by Prep-Cp.

Gene	Nucleotide Position	Amino Acid Position	Codon Conversion	Amino Acid Conversion
	473	158	tCa→tTa	S→L
ndhA	563	188	tCa→tTa	S→L
	1070	357	tCt→tTt	S→F
atpA	1148	383	tCa→tTa	S→L
atpB	1487	496	tCg→tTg	S→L
matK	1261	421	Cat→Tat	H→Y
	149	50	tCa→tTa	S→L
	467	156	cCa→cTa	P→L
	586	196	Cat→Tat	H→Y
ndhB	611	204	tCa→tTa	S→L
	704	235	tCc→tTc	S→F
	830	277	tCa→tTa	S→L
	836	279	tCa→tTa	S→L
	1481	494	cCa→cTa	P→L
ndhD	878	293	tCa→tTa	S→L
ndhF	62	21	tCa→tTa	S→L
	1487	496	aCg→aTg	T→M
petB	662	221	cCa→cTa	P→L
	2009	670	cCa→cTa	P→L
	2030	677	cCa→cTa	P→L
rpoC2	2158	720	Ccc→Tcc	P→S
	3002	1001	cCg→cTg	P→L
	4031	1344	tCg→tTg	S→L
rpl2	62	21	aCt→aTt	T→I
rpl20	308	103	tCa→tTa	S→L

rpoA	1009	337	Ctc→Ttc	L→F
rpoB	467	156	tCg→tTg	S→L
	545	182	tCa→tTa	S→L
	560	187	tCg→tTg	S→L
	617	206	cCg→cTg	P→L
rps8	182	61	tCa→tTa	S→L
ycf3	44	15	tCc→tTc	S→F
	191	64	aCg→aTg	T→M

Capitals in column Codon Conversion indicate target nucleotides.

Table S8. RNA editing sites in chloroplast genes of *Ae. speltoides* (AE918, TA1796 and PI487232) predicted by Prep-Cp.

Gene	Nucleotide Position	Amino Acid Position	Codon Conversion	Amino Acid Conversion
ndhA	473	158	tCa→tTa	S→L
	563	188	tCa→tTa	S→L
	1070	357	tCt→tTt	S→F
atpA	1148	383	tCa→tTa	S→L
atpB	35	12	gCt→gTt	A→V
atpB	1487	496	tCg→tTg	S→L
matK	1261	421	Cat→Tat	H→Y
ndhB	149	50	tCa→tTa	S→L
	467	156	cCa→cTa	P→L
ndhB	586	196	Cat→Tat	H→Y
	611	204	tCa→tTa	S→L
	704	235	tCc→tTc	S→F
	737	246	cCa→cTa	P→L
	830	277	tCa→tTa	S→L
	836	279	tCa→tTa	S→L
	1481	494	cCa→cTa	P→L
ndhD	878	293	tCa→tTa	S→L
ndhF	62	21	tCa→tTa	S→L
	1487	496	aCg→aTg	T→M
petB	662	221	cCa→cTa	P→L
rpoC2	2009	670	cCa→cTa	P→L
	2030	677	cCa→cTa	P→L
	2158	720	Ccc→Tcc	P→S
	3002	1001	cCg→cTg	P→L
	4031	1344	tCg→tTg	S→L
rpl2	62	21	aCt→aTt	T→I
rpl20	308	103	tCa→tTa	S→L
rpoA	1009	337	Ctc→Ttc	L→F
rpoB	467	156	tCg→tTg	S→L
	545	182	tCa→tTa	S→L
	560	187	tCg→tTg	S→L
	617	206	cCg→cTg	P→L
rps8	182	61	tCa→Ta	S→L
ycf3	44	15	tCc→tTc	S→F
	191	64	aCg→aTg	T→M

Capitals in column Codon Conversion indicate target nucleotides.

Table S9. RNA editing sites in chloroplast genes of *T. turgidum* ssp. *dicoccoides* (TA0073, TA0060 and TA1133) predicted by Prep-Cp.

Gene	Nucleotide Position	Amino Acid Position	Codon Conversion	Amino Acid Conversion
ndhA	473	158	tCa→tTa	S→L
	563	188	tCa→tTa	S→L
	1070	357	tCt→tTt	S→F
atpA	1148	383	tCa→tTa	S→L
atpB	35	12	gCt→gTt	A→V
	1487	496	tCg→tTg	S→L
matK	1261	421	Cat→Tat	H→Y
ndhB	149	50	tCa→tTa	S→L
	467	156	cCa→cTa	P→L
	586	196	Cat→Tat	H→Y
	611	204	tCa→tTa	S→L
	704	235	tCc→tTc	S→F
	737	246	cCa→cTa	P→L
	830	277	tCa→tTa	S→L
	836	279	tCa→tTa	S→L
	1481	494	cCa→cTa	P→L
ndhD	878	293	tCa→tTa	S→L
ndhF	62	21	tCa→tTa	S→L
	1487	496	aCg→aTg	T→M
petB	662	221	cCa→cTa	P→L
rpoC2	2009	670	cCa→cTa	P→L
	2030	677	cCa→cTa	P→L
	2158	720	Ccc→Tcc	P→S
	3002	1001	cCg→cTg	P→L
	4031	1344	tCg→tTg	S→L
rpl2	62	21	aCt→aTt	T→I
rpl20	308	103	tCa→tTa	S→L
rpoA	1009	337	Ctc→Ttc	L→F
rpoB	467	156	tCg→tTg	S→L
	545	182	tCa→tTa	S→L
	560	187	tCg→tTg	S→L
	617	206	cCg→cTg	P→L
rps8	182	61	tCa→tTa	S→L
ycf3	44	15	tCc→tTc	S→F
	191	64	aCg→aTg	T→M

Capitals in column Codon Conversion indicate target nucleotides.
